# Nitrogen application alleviates salt stress by enhancing osmotic balance, ROS scavenging, and photosynthesis of rapeseed seedlings (*Brassica napus*)

**DOI:** 10.1080/15592324.2022.2081419

**Published:** 2022-05-27

**Authors:** Tian Tian, Jingang Wang, Haijiang Wang, Jing Cui, Xiaoyan Shi, Jianghui Song, Weidi Li, Mingtao Zhong, Yue Qiu, Ting Xu

**Affiliations:** a College of Agriculture, Shihezi University, Xinjiang, China; b The Key Laboratory of Oasis Ecological Agriculture of Xinjiang Production and Construction Group, Shihezi University, Xinjiang, China

**Keywords:** Enzymes, non-enzymic antioxidants, organic osmoregulatory substance, inorganic ions, chlorophyll

## Abstract

Nitrogen application could alleviate salt stress on crops, but the specific physiological mechanism is still unclear. Therefore, in this study, a pot experiment was conducted to explore the effects of different application rates of nitrogen (0, 0.15, 0.30, and 0.45 g·kg^−1^) on the growth parameters, osmotic adjustment, reactive oxygen species scavenging, and photosynthesis of rapeseed seedlings planted in the soils with different concentrations of sodium chloride (1.5, 3.5, 5.5, and 7.5 g·kg^−1^). The results showed that nitrogen could alleviate the inhibition of salt on rapeseed growth, and improve the antioxidant enzyme activities and the contents of non-enzymatic substances, K^+^, soluble protein (SP), soluble sugar (SS), and proline. Besides, there was a significant correlation between the indexes of active oxygen scavenging system, osmoregulation system, and photosynthesis. Therefore, applying appropriate amount of nitrogen can promote the growth and development of rapeseed seedlings under salt stress, accelerate the scavenging of reactive oxygen species, maintain osmotic balance, and promote photosynthesis. This study will improve our understanding on the mechanism by which nitrogen application alleviates salt stress to crops.

## 1. Introduction

Salt stress is a major abiotic stress affecting the growth and development of crops.^1^ Due to excessive salt in the soil, a large number of chloride ions (Cl^−^) and sodium ions (Na^+^) penetrate into plant cells, destroy plant cells and organelles, affect plant metabolism, leading to the production and accumulation of toxic substances. Finally, crop cells are destroyed.^2^ High soil salinity have adverse effects on crop growth and development, nutrient absorption, photosynthesis, enzyme activity, protein synthesis, and hormone metabolism.^3^ Therefore, alleviating salt stress on crop physiological processes is a prerequisite to improve crop yield and quality.

Under salt stress, plants absorb a large number of salt ions, leading to the metabolic imbalance. To adapt to the high-salinity soil environment, crops often adjust their own osmotic balance, ion distribution, reactive oxygen species (ROS), photosynthesis, and other physiological processes,^4^ to reduce the toxicity of salt to their growth and development. Some studies have shown that crops respond to salt stress by reducing the osmotic potential of cells and enhancing the water absorption capacity of roots by regulating inorganic ions^5^ or synthesizing and accumulating organic substances^6^ in cytoplasm, to alleviate the osmotic stress caused by salt. Besides, under salt stress, crops increase the activity of antioxidant enzymes^7^ and the content of small molecular antioxidants (ascorbic acid,^8^ glutathione,^9^ tocopherol,^10^ etc.), to reduce the damage caused by excessive ROS, such as membrane damage, protein degradation, and enzyme inactivation, and alleviate the toxicity of soil salt. Sun et al.^11^ found that when the salt concentration was 0 ~ 100 mmol·L^−1^, the stomatal opening was reduced in cotton seedlings, to maintain the photosynthetic organs undamaged and normal photosynthesis. However, the self regulation ability of crop to salt stress has certain limitations. Excessive soil salt always causes osmotic stress, ion toxicity, and photosynthetic damage to crops and affects crop growth and development.^12^

Nitrogen is one of the essential elements for crop growth and development. It is a component of the main substances in crops, such as nucleic acid, protein, and chloroplast.^13^ Nitrogen actively participates in a variety of physiological pathways, such as osmotic substance synthesis,^14^ ROS scavenging,^15^ photosynthesis regulation,^16^ and so on. Ahanger et al.^17^ pointed out that nitrogen application could enhance the accumulation of antioxidant substances and osmolyte metabolism of wheat, and alleviate the growth inhibition caused by salt stress. Huang et al.^18^ showed that compared with low nitrogen supply, high nitrogen supply could maintain constant leaf water potential and stomatal conductance (g_s_) by improving rice leaf hydraulic conductivity, and significantly improve photosynthesis and tolerance to high temperature stress. Alla et al.^19^ showed that nitrogen application could improve the photosynthetic activity of maize seedlings under NaCl stress, inhibit the reduction of protein and pigments, and effectively reduce the impact of salt stress. Gao et al.^20^ showed that nitrogen application could increase the content of antioxidants and the activity of antioxidant enzymes in *Nitraria japonica* under salt stress, and activate the ascorbic acid-glutathione (ASA-GSH) cycle through improving the expression of related genes, so as to eliminate excessive reactive oxygen species and alleviate salt stress.

Rapeseed has the characteristics of low planting cost, high yield, and high nutritional value,^21^ and can improve soil physical and chemical properties. However, rapeseed is sensitive to soil salt at seedling stage. Excessive soil salt often causes water loss and ion toxicity, and destroys normal metabolic function of plants.^22^ However, at present, the effect of nitrogen application on osmotic stress, ion toxicity, and photosynthetic damage caused by salt is not clear. There, this study (1) explored the effects of salt stress on the growth and physiological indexes of rapeseed seedlings, (2) determined the effect of nitrogen application on osmotic regulation and ROS scavenging system of rapeseed seedlings under salt stress, and (3) clarified the response mechanism of physiological and biochemical indexes and photosynthesis of rapeseed seedlings to nitrogen application under salt stress. This study will improve our understanding on the mechanism by which nitrogen application alleviates salt stress to crops.

## 2. Materials and methods

### 2.1. Study site

The experiment was conducted at the Experimental Station of Shihezi University, Xinjiang Uygur Autonomous Region, China (86°3′N, 44°18′E, 428 m a.s.l.) in 2020. The region has a temperate continental climate, with an annual sunshine duration of 2,725–2,820 h, an accumulated temperature ≥10°C of 3,595–3,729°C, an annual precipitation of 125.0–207.7 mm, and a frost-free period of 168–171 days. The soil was sandy loam, with pH value of 7.68, organic matter of 11.25 g·kg^−1^, total nitrogen of 0.89 g·kg^−1^, available phosphorus of 18.70 mg·kg^−1^, available potassium of 242.00 mg·kg^−1^, and salinity of 0.53 g·kg^−1^ .

### 2.2. Experimental design

**Figure 1.**
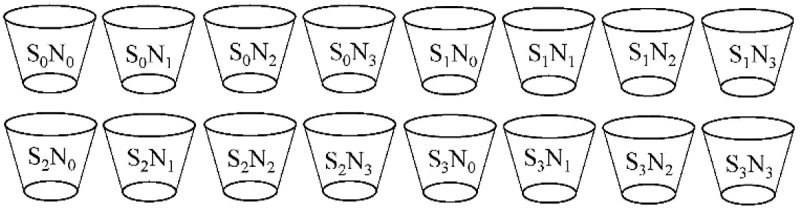
Schematic diagram of potted plant layout under different treatments.

Note: a – change in SP content, b – change in SS content, c – change in Pro content, d – change in K^+^ content, e – change in Na^+^ content

The experiment employed a random block design with two factors (NaCl application and nitrogen application). Four gradients of salt application rate (0 (S_0_), 3.5 (S_1_), 5.5 (S_2_), and 7.5 (S_3_) g·kg^−1^) and four nitrogen application rates (0 (N_0_), 0.15 (N_1_), 0.30 (N_2_), and 0.45 (N_3_) g·kg^−1^) were set up. There were a total of 16 treatments, and each treatment had 10 replicates (Figure 1).

On July 25th, rapeseed seeds (variety: Huayouza No. 62) were sown in plastic trays containing soil and vermiculite mixture (V:V = 1:1) at a depth of 3–5 cm (one seed per tray). Then, the trays were transferred into an incubator with light intensity of 7,500–8,500 lx, temperature of 15–20°C, and light time of 12–14 h per day, and sprayed with deionized water daily to keep the soil surface moist. Different amounts of salt and nitrogen were mixed evenly with the soil collected from the field (0–20 cm soil layer) separately and transferred in pots (upper diameter: 25 cm, bottom diameter: 20 cm, height: 30 cm, soil weight: 13 kg). When most seedlings had three leaves, the seedlings with uniform growth were selected. The soils on the roots of the selected seedlings were cleaned before transplanting into pots. Three seedlings were planted in one pot. Concentrated superphosphate (P_2_O_5_, 46–54%) of 0.25 g·kg^−1^ and potassium sulfate (K_2_O, 50%) of 0.15 g·kg^−1^ were applied once for each pot, and 500 mL of water was applied to each pot during each irrigation to prevent leakage of salt from pots. Other managements were consistent. On August 29, one plant was sampled from one pot. The samples were put into sealed bags, wrapped with tine foil, ice-cooled in an box, and taken back to the lab for the physiological determinations.

### 2.3. Determination methods

#### 2.3.1 Determination of growth parameters

Rapeseed seedlings were collected from each treatment. Then, the seedlings were dried to constant weight, and weighed to calculate the average dry weight for each treatment. The leaf area was measured by leaf area meter (resolution: 1 mm^2^, LI-COR LI-3100C, USA). Water content (%) was determined according to the following formula

$$Water\;content\left(\%\right)=\left(FW-DW\right)/FW\times 100\%$$

Where FW is the fresh weight of sample, and DW is the dry weight of sample.

#### 2.3.2 Determination of indexes of ROS scavenging system in rapeseed seedlings

Fresh rapeseed sample (0.50 g) was ground in ice bath with 5 mL phosphate buffer (pH: 7.8) in a mortar, then the enzyme solution was transferred into test tube after freezing and centrifugation, and stored at 0–4°C. The superoxide dismutase (SOD) activity was determined according to Wei et al. (2021). SOD reaction solution consisting of tetrazolium blue, methionine, riboflavin, and EDTA-Na_2_, was mixed with 200 μL enzyme solution, and the absorbance was measured at 560 nm. The peroxidase (POD) activity was determined according to Wei et al. Enzyme solution (100 μL) and 3 mL of POD guaiacol reaction solution were transferred in a cuvette. Then, the absorbance was measured once every 1 min at 470 nm, and a total of three-time measurement was conducted. The enzyme activity was expressed with the absorbance change value per min. The catalase (CAT) activity was measured according to Wei et al. Three milliliters of 20 mmol∙L^−1^ H_2_O_2_ solution was mixed with 100 μL enzyme extract, and then the absorbance was measured at 240 nm after 15s. The data were obtained every 30s and a total of 6 time data acquisition was conducted. The malonaldehyde (MDA) content was determined according to Wei et al. Enzyme extract (1 mL) was diluted with 1 mL of phosphoric acid buffer (pH: 7.8), then 4 mL of thiobarbituric acid solution was added, boiled for 15 min, and then cooled rapidly after centrifugation. The absorbance was determined at 450, 532, and 600 nm.^23^

The content of ascorbic acid (ASA) was determined by spectrophotometer.^24^ Fresh rape seedlings (5.00 g) was ground into slurry with 20 mL of 50 g∙L^−1^ trichloroacetic acid solution under the condition of ice bath, and then the volume was made up to 100 mL with 50 g∙L^−1^ trichloroacetic acid solution, followed by an extract for 10 min. Sample extract (1 mL) was added into the test tube, followed by the addition of 1 mL of trichloroacetic acid solution, 1 mL of absolute ethanol, 0.5 mL of 0.4% phosphoric acid ethanol solution, 1 mL of BP-ethanol solution, and 1 mL of FeCl_3_-ethanol solution. Then, the absorbance was determined at 534 nm.^24^ The content of reduced glutathione (GSH) was determined according to Li et al. Fresh rapeseed seedlings (2.50 g) was ground into homogenate with 5 mL of 50 g∙L^−1^ trichloroacetic acid solution under the condition of ice bath, and the supernatant was collected after centrifugation. Then, 1 mL of distilled water, 1 mL of 0.1 mol∙L^−1^ phosphoric acid buffer (pH = 7.7), and 0.5 mL of 4 mmol∙L^−1^ DTNB solution were added into a test tube and mixed well. This solution was taken as a reference to zero the spectrophotometer at 412 nm. Another two test tubes were prepared and 1 mL of supernatant and 1 mL of phosphate buffer (pH: 7.7) were added respectively. Then, 0.5 mL of 4 mmol∙L^−1^ DTNB solution was added into one test tube and 0.5 mL of phosphoric acid buffer (pH: 6.8) was added into the other test tube. The two tubes were kept at 25°C for 10 min, and the absorbance was measured at 412 nm.^24^ The content of carotenoid (Car) was determined according to Ahanger et al. Fresh rapeseed leaves (0.2 g) was transferred in a 10 mL volumetric flask, shaken well after adding 10 mL of extraction solution (acetone: ethanol = 1:1), and stored in dark overnight. After the leaves were completely white, the absorbance was measured at 470 nm.^17^

#### 2.3.3 Determination of indexes of osmotic regulation system of rapeseed seedlings

The relative conductivity was measured by conductivity meter (Mettler-Toledo, Switzerland). Fresh rapeseed leaves were washed with distilled water for 1 ~ 2 times. Then, 0.20 g leaf samples were cut into pieces, and immersed in 20 mL of distilled water in a test tube with plug at room temperature for 5 h. After that, the conductivity was measured with the S230-K-CN conductivity meter. The conductivity was measured again after a 15-min boil water bath and cooling to room temperature.^23^

The content of soluble protein (SP) was determined by Coomassie brilliant blue method.^23^ Fresh rapeseed sample (0.25 g) was ground into homogenate with 5 mL distilled water and centrifuged. Then, the supernatant (0.3 mL) was mixed with 5 mL Coomassie brilliant blue reagent in the test tube and shaken well. The absorbance was measured at 595 nm after 2 min.^23^ The content of soluble sugar (SS) was determined according to Li et al.^25^ Fresh rapeseed sample (0.10 g) was cut into pieces and 5 mL distilled water was added. Then, the mouth was sealed with sealing film, followed by an extraction in boiling water for 30 min. This step was repeated twice. The extract was filtered to a constant volume of 25 mL. After that, 0.5 mL of extract was transferred into a test tube, and 1.5 mL distilled water, 1 mL 9% phenol solution, and 5 mL concentrated sulfuric acid were added. The tube was shaken and place at room temperature for 30 min, Colorimetry was performed at the wavelength of 485 nm.^25^ The content of proline (Pro) was determined according to Li et al.^25^ Fresh rapeseed sample (0.10 g) was mixed with 3 mL of 5% sulfosalicylic acid and extracted in boiling water bath for 10 min. The filtrate was collected after cooling (proline extract). Then, 2 mL extract, 2 mL glacial acetic acid, and 2 mL acid ninhydrin test agent were added into a covered test tube, followed by a boiling water bath for 30 min. After that, 4 mL toluene was added after cooling, followed by shaking for 30s. The upper solution was pipetted into a cuvette and the absorbance was measured at 520 nm.^25^

The contents of potassium (K^+^) and sodium (Na^+^) were determined according to Guo et al.^26^ Dried rapeseed seedlings (0.5 g) was mixed with 10 mL deionized water, followed by a water bath for 2 h. Then, 5 mL was absorbed, and the volume was made up to 50 mL with ionic water. Finally, the contents of potassium (K^+^) and sodium (Na^+^) were determined with the flame photometer (FP640, Shanghai, China).^26^

#### 2.3.4 Determination of photosynthetic indexes of rapeseed seedlings

The chlorophyll content (Chl) was measured by spectrophotometer method.^27^ Fresh rapeseed leaves (0.2 g) were transferred in a 10 mL volumetric flask, and 10 mL of extraction solution (acetone: ethanol = 1:1) was added. Then, the flask was placed in dark at room temperature after fully shaking. After the leaves were completely white, the absorbance were measured at 665 and 649 nm. The fully expanded leaves were selected to measure the photosynthetic parameters from 9:00 to 11:00. The net photosynthetic rate (P_n_), stomatal conductance (g_s_), and transpiration rate (T_r_) were measured at the same leaf position of the same plant by Li-6400 portable photosynthetic instrument (li-cor, Lincoln, NE, USA). The light intensity of the portable photosynthetic determination system was set as 1000 µmol·m^−2^s^−1^, the CO_2_ concentration was 400 µmol·mol^−1^, and the temperature was 25°C.^27^

### 2.4 Data Processing

Excel 2013 (Microsoft Office, Redmond, USA) and SPSS 19.0 (Statistical Product and Service Solutions, Chicago, USA) software were used for data processing and Pearson analysis. Significant differences were determined using Duncan’s multiple comparisons (*P* < .05). Origin 2018 (Origin Lab, Massachusetts, USA) and PowerPoint 2016 (Microsoft Office, Redmond, USA) were used to create the Figures

## 3. Results

### 3.1. Effect of salt and nitrogen application on the growth of rapeseed seedlings

Salt application significantly suppressed the growth of rapeseed seedlings (Table 1). With the increase of soil salinity, the relative conductivity and MDA content increased significantly (*P* < .05), while the dry weight (*P* > .05), leaf area (*P* < .05), and water content (*P* > .05) decreased. The S_3_N_0_ treatment had the most significant changes. The relative conductivity and MDA content in the S_3_N_0_ treatment increased by 48.34% and 43.34%, respectively (*P* < .05), and the dry weight, leaf area, and water content decreased by 19.49%, 18.28%, and 7.21%, respectively (*P* < .05).

**Table 1.** Effects of nitrogen application on the growth of rapeseed seedlings at different levels of soil salinity

Treatment		Dry weight (g)		Leaf area (cm^2^)	Water content (%)	Relative conductivity (%)	MDA content (nmol·g^−1^)
S_0_	N_0_	1.46 ± 0.09cB		12.17 ± 0.18deC	91.11 ± 0.82abcA	45.11 ± 2.59efA	8.80 ± 0.23 dA
	N_1_	1.49 ± 0.03cB		12.81 ± 0.20cC	93.40 ± 0.67abA	44.05 ± 2.64fA	6.76 ± 0.42efB
	N_2_	2.10 ± 0.05aA		14.53 ± 0.22aA	95.00 ± 2.28aA	36.36 ± 2.55gB	5.95 ± 0.29fC
	N_3_	1.69 ± 0.16bB		13.18 ± 0.10cB	93.75 ± 2.90abA	43.01 ± 2.55fA	6.18 ± 0.21efBC
S_1_	N_0_	1.22 ± 0.09 dB		11.95 ± 0.18deC	89.82 ± 3.00abcdA	50.10 ± 2.76eA	8.25 ± 0.19 dA
	N_1_	1.31 ± 0.09cdAB		11.98 ± 0.07deC	89.90 ± 3.40abcdA	49.99 ± 2.68eA	8.20 ± 0.17 dA
	N_2_	1.50 ± 0.10cA		13.91 ± 0.25 bA	90.84 ± 2.44abcA	44.69 ± 3.38A	6.00 ± 0.30fC
	N_3_	1.46 ± 0.12cAB		13.14 ± 0.32cB	90.45 ± 2.49abcdA	46.43 ± 1.72efA	6.70 ± 0.23efB
S_2_	N_0_	1.20 ± 0.11 dB		11.69 ± 0.35eA	86.84 ± 2.53cdA	59.07 ± 2.67 dA	10.81 ± 0.75cA
	N_1_	1.29 ± 0.06cdAB		10.81 ± 0.21fB	86.96 ± 1.92cdA	58.14 ± 2.92 dA	8.33 ± 0.25 dB
	N_2_	1.47 ± 0.08cA		12.34 ± 0.29 dA	88.33 ± 3.39bcdA	57.12 ± 2.71 dA	7.08 ± 0.20eC
	N_3_	1.38 ± 0.06cdAB		11.95 ± 0.23deA	87.76 ± 2.05bcdA	57.71 ± 1.82 dA	10.61 ± 0.46cA
S_3_	N_0_	1.18 ± 0.080 dA		9.79 ± 0.28gC	84.55 ± 2.94 dA	87.32 ± 2.36aA	12.62 ± 0.55aA
	N_1_	1.19 ± 0.09 dA		10.69 ± 0.21fB	85.14 ± 2.09cdA	74.12 ± 2.58bB	11.88 ± 0.61abAB
	N_2_	1.25 ± 0.06 dA		11.77 ± 0.25eA	86.49 ± 2.72cdA	66.30 ± 2.40cC	10.89 ± 0.58cB
	N_3_	1.24 ± 0.11 dA		10.75 ± 0.17fB	85.19 ± 3.39cdA	72.11 ± 2.37bB	11.22 ± 0.75bcAB
Significance (*P-*value)	Salt treatment (ST)		**	**	**	**	**
	Nitrogen treatment (NT)		**	**	ns	**	**
	ST×NT		**	**	ns	**	**

Note: Different lowercase letters in the same column indicate significant difference between treatments (*P* < 0.05), and different uppercase letters in the same column indicate significant difference between nitrogen treatments at the same salinity level (*P* < 0.05). The same below.

Nitrogen application had different effects on the relative conductivity, MDA content, dry weight, leaf area, and water content in rapeseed seedlings at different levels of soil salinity. There were obvious changes in the above indexes in the S_1_N_2_ and S_2_N_2_ treatments compared to the S_1_N_0_ and S_2_N_0_ treatments, respectively (*P* < .05), while the changes in MDA content and water content were not obvious in the S_3_N_1_, S_3_N_2_, and S_3_N_3_ treatments. Compared to S_1_N_0_ treatment, S_1_N_2_ treatment had the lowest relative conductivity (49.99%) and MDA content (8.20 nmol·g^−1^) and the highest dry weight (1.50 g), leaf area (13.91 cm^2^), and water content (90.84%). The relative conductivity and MDA in the S_1_N_3_ treatment increased by 3.75% and 10.31%, respectively, and the dry weight, leaf area, and water content decreased by 2.41%, 5.56%, and 0.43%, respectively compared with those in the S_1_N_2_ treatment. According to the results of two-factor ANOVA analysis, the effects of salt application, nitrogen application, and their interaction on dry weight, leaf area, relative conductivity, and MDA content reached a significant level.

*3.2. Effects of nitrogen application on the osmoregulatory system of rapeseed seedlings at different levels of soil salinity*

**Figure 2.**
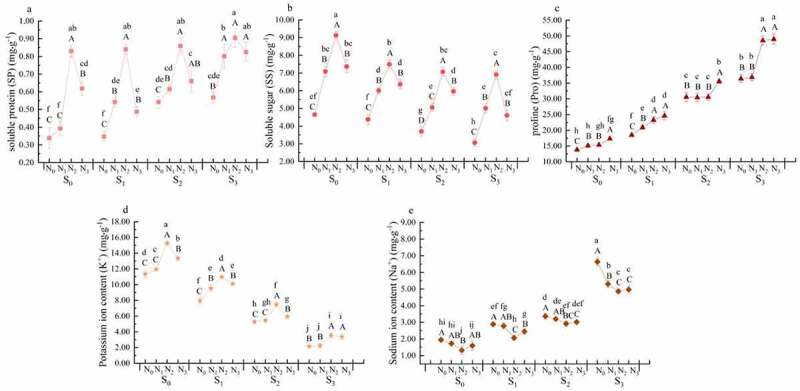
Effects of nitrogen application on inorganic ions and organic osmotic regulatory substances in the osmotic regulation system of rape seedlings under different salinity content.

Note: a – change in POD activity, b – change in SOD activity, c – change in CAT activity, d – change in ASA content, e – change in GSH content, f – change in Car content

The content of Pro increased with the increase of soil salinity (*P* < .05) (Figure 2). The content of Pro in the S_3_N_0_ treatment increased by 163.60% compared with that in the S_0_N_0_ treatment (*P* < .05). The content of SP in the S_2_N_0_ and S_3_N_0_ treatments increased by 37.45% and 40.32%, respectively (*P* < .05) compared with that in the S_0_N_0_ treatment. The content of SS in the S_2_N_0_ and S_3_N_0_ treatments decreased by 20.52% and 34.07%, respectively, compared with that in the S_0_N_0_ treatment (*P* < .05). The content of Na^+^ increased but the content of K^+^ decreased, with the increase of soil salinity (*P* < .05). The S_3_N_0_ treatment had the highest content of Na^+^ (7.63 mg·g^−1^) and the lowest content of K^+^ (2.16 mg·g^−1^).

Nitrogen application increased the content of SP, SS, Pro, and K^+^, but decreased the content of Na^+^ at all levels of soil salinity (*P* < .05). At the same salinity level, the content of SP, SS, and K^+^ increased first and then decreased with the increase of nitrogen application rate, and the highest content of SP, SS, and K^+^ were observed in the N_2_ treatments. The content of SP, SS, and K^+^ in the S_1_N_2_ treatment increased by 142.08%, 71.17%, and 37.97%, respectively (*P* < .05), compared with those in the S_1_N_0_ treatment, while the content of SP, SS, and K^+^ in the S_1_N_3_ treatment decreased by 42.02%, 15.09%, and 87.90% (*P* < .05), respectively, compared with those in the S_1_N_2_ treatment. Same trends were observed at S_2_ and S_3_ levels. The content of Pro showed an increasing trend with the increase of nitrogen application rate at the same salinity level. The content of Pro in the S_1_N_1_, S_1_N_2_, and S_1_N_3_ treatments all increased (*P* < .05). There was no difference between S_2_N_1_ and S_2_N_0_ treatments and between S_3_N_1_ and S_3_N_0_ treatments, but the content of Pro in the N_3_ treatments were higher than that in the N_0_ treatments (*P* < .05). The content of Na^+^ decreased and then increased with the increase of nitrogen application rate, and the lowest was found in the N_2_ treatments. The Na^+^ content in the S_1_N_2_ and S_1_N_3_ treatments decreased by 28.44% and 15.09%, respectively (*P* < .05) compared with that in the S_1_N_0_ treatment, and the Na^+^ content in the S_2_N_2_ and S_2_N_3_ treatments decreased by 13.39% and 10.51%, respectively compared with that in the S_2_N_0_ treatment (*P* < .05). The content of Na^+^ in the S_3_N_1_, S_3_N_2_, and S_3_N_3_ treatment decreased (*P* < .05) compared with that in the S_3_N_0_ treatment, while the content of Na^+^ in the S_1_N_3_, S_2_N_3_, and S_3_N_3_ treatments increased by 17.16%, 15.73%, and 2.57%, respectively (*P* < .05), compared with that in the S_1_N_2_, S_2_N_2_, and S_3_N_2_ treatments.

*3.3. Effects of nitrogen application on the ROS scavenging system of rapeseed seedlings at different levels of soil salinity*

**Figure 3.**
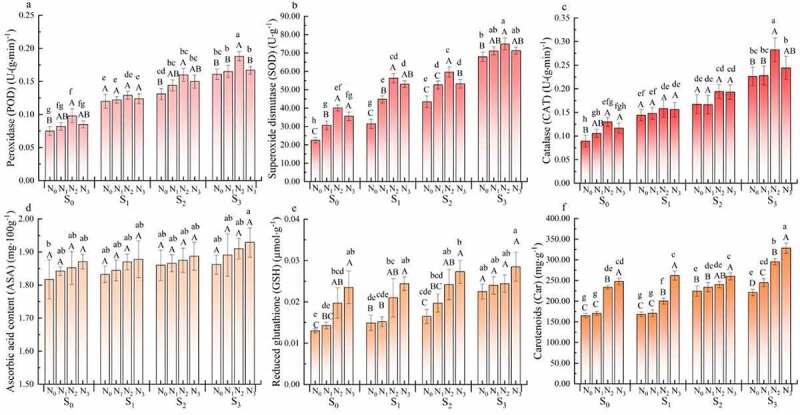
Effects of nitrogen application on enzymatic and non-enzymatic lines in the ROS clearance system of rape seedlings under different salt content. Note: Light color line indicates insignificant correlation, virtual line indicates significant correlation (* indicates significance at 0.05 level), and real line indicates extremely significant correlation (** indicates significance at 0.01 level)

The activities of antioxidant enzymes (POD, SOD, and CAT) and the contents of non-enzymatic substances (ASA, GSH and Car) in rapeseed seedlings increased with the increase of soil salinity (Figure 3). The activities of POD, SOD, and CAT and the contents of ASA, GSH, and Car in the S_3_N_0_ treatment were the highest among the treatments, which were 0.16 U·(g·min)^−1^, 67.97 U·g^−1^, 0.23 U·(g·min)^−1^, 1.86 mg·100 g^−1^, 0.02 µmol·g^−1^, and 220.98 mg·g^−1^, respectively.

Nitrogen application increased the antioxidant enzyme activity and the content of non-enzymatic substances in rapeseed seedlings at different levels of soil salinity (*P* < .05). Whether at the same salinity level or at different salinity levels, the variation trends of antioxidant enzyme activity were the same. The antioxidant enzyme activity increased with the increase of nitrogen application rate and the N_2_ treatments had the highest antioxidant enzyme activity. The POD, SOD, and CAT activities in the S_1_N_2_ treatment increased by 6.98%, 44.10%, and 8.86%, respectively, compared with those in the S_1_N_0_ treatment. However, the POD, SOD, and CAT activities in the S_1_N_3_ treatment decreased by 3.88%, 24.22%, and 1.27%, respectively compared with those in the S_1_N_2_ treatment. At the same soil salinity, the contents of GSH and Car increased with the increase of nitrogen application rate (*P* < .05). The ASA content also increased (*P* > .05), and the highest was found in the N_3_ treatments. The contents of ASA, GSH, and Car in the S_1_N_3_ treatment increased by 2.45%, 37.50%, and 35.88%, respectively, compared with those in the S_1_N_0_ treatment.

*3.4. Effects of nitrogen application on photosynthesis of rapeseed seedlings at different levels of soil salinity*

Soil salinity suppressed the synthesis of chlorophyll in rapeseed seedlings (Table 2). The chlorophyll content, photosynthetic rate, P_n_, g_s_, and Tr all decreased with the increase of soil salinity. There were differences in the Chl, P_n_, g_s_, and Tr between S_3_ and S_0_ treatments (*P* < .05). The Chl content, P_n_, g_s_, and Tr were the lowest in the S_3_N_0_ treatment, decreasing by 36.60%, 33.33%, 12.53%, and 15.67%, respectively, compared with those in the S_0_N_0_ treatment.

**Table 2.** Effects of nitrogen application on photosynthesis of rapeseed seedlings at different levels of soil salinity

Treatment		Total chlorophyll (Chl) (μg·g^−1^)	Photosynthetic rate (P_n_) (µmol·m^−2^·s^−1^)	Stomatal conductance (g_s_) (mmol·m^−2^·s^−1^)	Transpiration rate (T_r_) (mmol·m^−2^·s^−1^)
S_0_	N_0_	1756.80 ± 51.51cdC	18.10 ± 0.72bcdeB	0.77 ± 0.02fgC	4.00 ± 0.13defD
	N_1_	1949.26 ± 127.91bcBC	19.37 ± 0.85abcAB	1.13 ± 0.08cB	4.62 ± 0.11cdC
	N_2_	2508.32 ± 66.95aA	20.73 ± 0.85aA	1.55 ± 0.06aA	6.38 ± 0.15aA
	N_3_	2122.13 ± 206.72bB	18.37 ± 1.06bcdeB	1.15 ± 0.04cB	5.78 ± 0.26abB
S_1_	N_0_	1640.45 ± 63.08deA	17.33 ± 0.78deA	0.76 ± 0.01gC	3.93 ± 0.26efB
	N_1_	1669.57 ± 153.92deA	17.64 ± 1.22cdeA	1.00 ± 0.03 dB	4.67 ± 0.57cB
	N_2_	1743.20 ± 171.71cdA	19.67 ± 0.57abA	1.35 ± 7.34 bA	5.91 ± 0.15abA
	N_3_	1716.67 ± 42.21cdeA	17.43 ± 1.67deA	1.30 ± 0.03 bA	4.61 ± 0.36cB
S_2_	N_0_	1455.63 ± 200.29efA	14.63 ± 0.76fC	0.76 ± 0.02gC	3.56 ± 0.51fC
	N_1_	1538.85 ± 72.64deA	17.40 ± 0.47deB	0.82 ± 0.01efgC	4.54 ± 0.35cdeB
	N_2_	1602.97 ± 30.52deA	18.60 ± 0.24bcdA	1.03 ± 0.02 dA	5.84 ± 0.240abA
	N_3_	1542.89 ± 79.68deA	16.67 ± 0.47eB	0.89 ± 0.03eB	4.75 ± 0.07cB
S_3_	N_0_	1113.86 ± 91.39 gA	12.07 ± 0.17gC	0.67 ± 0.03hD	3.37 ± 0.21fC
	N_1_	1132.07 ± 130.24 gA	17.40 ± 0.42deB	0.85 ± 0.036efC	3.87 ± 0.13fC
	N_2_	1257.37 ± 51.92fgA	18.57 ± 0.42bcdA	1.03 ± 0.03 dA	5.52 ± 0.35 bA
	N_3_	1146.03 ± 122.77 gA	16.73 ± 0.37deB	0.90 ± 0.02eB	4.66 ± 0.07cB
Significance (*P-*value)	Salt treatment (ST)	**	**	**	**
	Nitrogen treatment (NT)	**	**	**	**
	ST×NT	**	**	**	**

Nitrogen application increased Chl content, P_n_, g_s_, and T_r_ (*P* < .05) at all soil salinity levels except for the P_n_ at S_1_ level. The variation trends of Chl content, P_n_, g_s_, and T_r_ in the S_2_ and S_3_ treatments were the same as those in the S_1_ treatments. The Chl, P_n_, g_s_ and T_r_ were the highest in the S_1_N_2_ treatment, increasing by 5.89%, 11.87%, 43.93%, and 33.52%, respectively, while those in the S_1_N_3_ treatment decreased by 1.52%, 11.36%, 3.95%, and 21.93%, respectively, compared with those in the S_1_N_0_ treatment. The two-factor ANOVA showed that the effects of salt application, nitrogen application, and their interaction on Chl, P_n_, g_s_, and T_r_ all reached significant levels.

### 3.5. Correlation analysis of ROS scavenging system and osmoregulatory system in rapeseed seedlings

**Figure 4.**
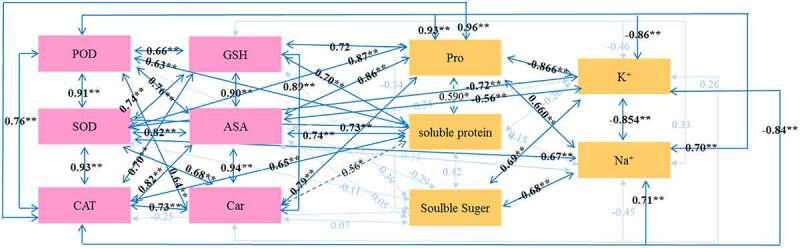
Schematic diagram of the correlation between the indicators of the ROS removal system and the penetration regulation system.

Note: * represents significance at 0.05 level, ** represents significance at 0.01 level

There was a positive correlation between the enzymes (POD, SOD, and CAT) and the non-enzymatic substances (GSH, ASA, and Car) in the ROS scavenging system (*P* < .01) (Figure 4). The highest correlation was found between ASA and Car (correlation coefficient: 0.94), followed by the correlation between POD and Car (0.64). The correlations between organic osmoregulatory substances were low. However, there was a negative correlation between K^+^ and Na^+^ (−0.85) (*P* < .01). The Pro had a negative correlation with K^+^ (−0.87) and a positive correlation with Na^+^ (0.66). The SS had a positive correlation with K^+^ (0.69) and a negative correlation with Na^+^ (−0.68) (*P* < .01).

There was a correlation between the substances in the ROS scavenging system and the substances in the osmoregulatory system (*P* < .05). In comparison with organic osmoregulatory substances, the enzymes (POD, SOD, and CAT) and non-enzymatic substances (GSH, ASA, and Car) in the ROS scavenging system all had positive correlations with Pro (*P* < .01), and the highest correlation was found between CAT and Pro (0.96). The enzymes (POD, SOD, and CAT) and non-enzymatic substances (GSH and ASA) had positive correlations with SP (*P* < .01), and the highest correlation was found between ASA and SP (0.74). Car had a positive correlation with SP (0.56) (*P* < .05). In comparison with inorganic osmoregulatory substances, the enzymes (POD, SOD, and CAT) and ASA had negative correlations with K^+^ (*P* < .05), and the highest correlation was found between CAT and K^+^ (−0.84). The enzymes had positive correlations with Na^+^ (*P* < .05), and the highest correlation was found between Na^+^ and CAT (0.71), while the non-enzyme substances (GSH, ASA, and Car) had lower correlations with Na^+^.

*3.6. Correlation analysis of photosynthetic parameters with the substances in ROS scavenging system and osmoregulatory system in rapeseed seedlings*

**Figure 5.**
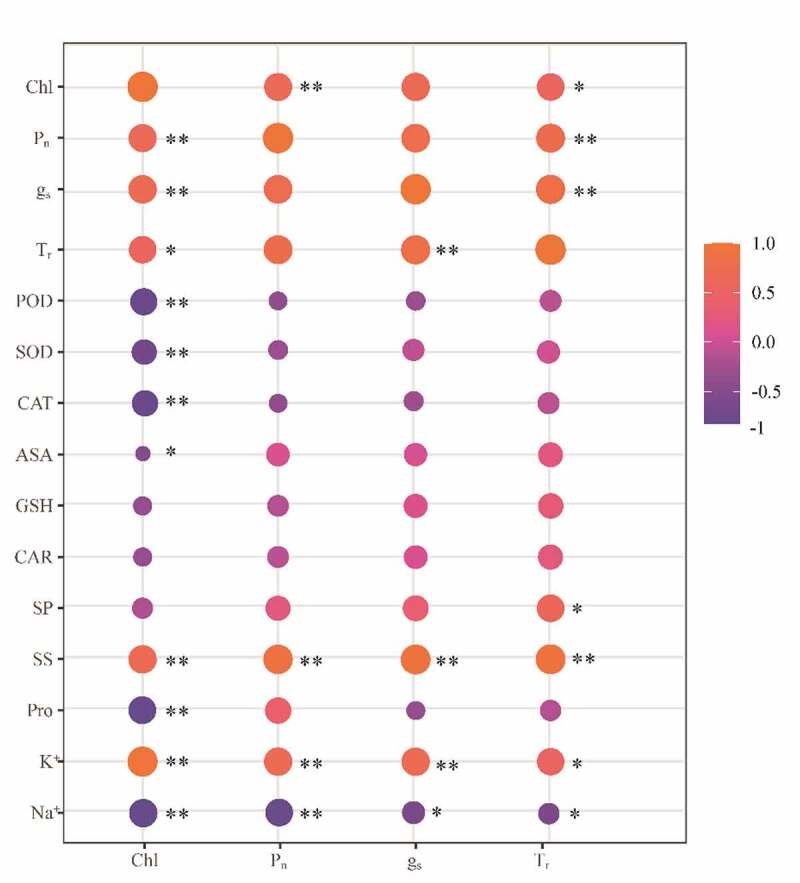
Schematic diagram of physiological indicators significantly related to photosynthesis and ROS removal system and osmotic regulation system.

Chl had a positive correlation with P_n_, g_s_, and T_r_ (*P* < .05) (Figure 5). The physiological indicators with high correlations with Chl, P_n_, g_s_, and T_r_ were screened in this study. Chl had a positive correlation with P_n_, g_s_, and T_r_, with correlation coefficient of 0.65, 0.70, and 0.58, respectively. Chl had a negative correlation with POD, SOD, and CAT in the ROS scavenging system (*P* < .01). Among them, the correlation between Chl and POD was the highest (−0.81). Chl had a negative correlation with ASA (−0.50) (*P* < .05). Chl had a positive correlation with SS and K^+^ (0.70 and 0.96) (*P* < .01), and a negative correlation with Pro (−0.82) (*P* < .01). P_n_, g_s_, and T_r_ had a positive correlation with SS and K^+^ (*P* < .05), and the correlation between T_r_ and SS was the highest (0.93). P_n_ had a negative correlation with Na^+^ (−0.80) (*P* < .01), and g_s_ and T_r_ had negative correlations with Na^+^ (−0.62 and −0.589, respectively) (*P* < .05).

## 4. Discussion

**Figure 6.**
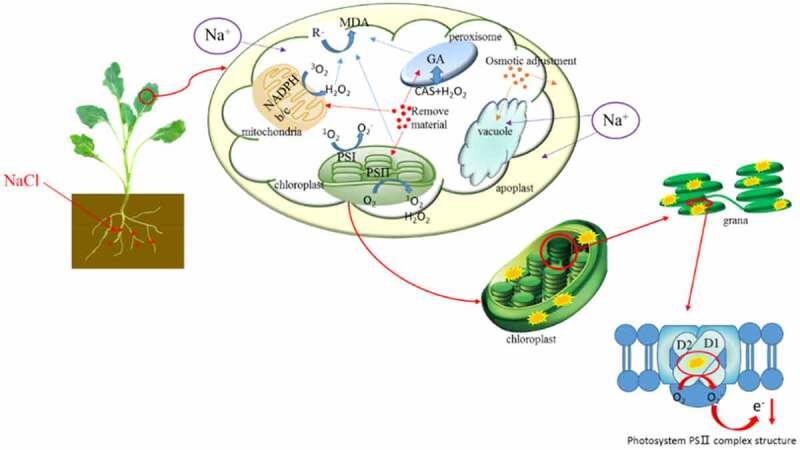
Schematic diagram of the physiological effects of soil salinity on rapeseed seedlings.

The effects of soil salinity on crops are multifaceted. When the salt accumulated in crops reaches a certain amount, various physiological and biochemical reactions and even growth of crops will be hindered.^28^ In this study, it was found (Figure 6) that with the increase of soil salinity, a large amount of Na^+^ accumulated in rapeseed seedlings, and the relative conductivity increased, resulting in the decrease of osmotic potential and osmotic stress. It may be due to the increased permeability of plasma membrane and the volume reduction and water loss in cytoplasm, vesicles, and other parts containing tissue fluid.^29^ The increase of MDA content may be due to that excessive accumulation of ROS in organelles such as mitochondria and peroxisomes leads to the breaking of balance and the damage to membrane integrity and biological functional molecules such as nucleic acids, proteins, and lipids.^30^ In addition, in our study, salt stress led to decreased chlorophyll content, P_n_, g_s_, and T_r_, and weakened photosynthesis. This may be due to that excessive accumulation of reactive oxygen species (ROS) in photosystems I and II induced by salt stress distorts, loosens, and damages the thylakoid lamellae of chloroplasts, and destroys enzymes and proteins involved in light reactions and the Calvin cycle.^31^ At different levels of soil salinity, a series of physiological and biochemical responses (osmotic adjustment and ROS scavenging) were found in rapeseed seedlings to temporarily alleviate the salt stress. On the one hand, rapeseed seedlings improve their tolerance by accumulating osmoregulatory substances (SP and Pro). On the other hand, the SOD, POD, and CAT activities are enhanced, and the accumulation of non-enzymatic substances (GSH and Car) is increased to alleviate the toxic effects generated by ROS by scavenging superoxide anion free radical, hydrogen peroxide, and hydroxyl radicals.^12,32^ The contents of SS and K^+^ in rapeseed seedlings reduced at different levels of soil salinity in this study, which was consistent with the results of Mansour et al.^26^ However, Zhao et al.^33^ found that the content of SS increased with the increasing of soil salinity. It may be due to that the content of SS, one of the initial products of photosynthesis,^34^ could be reduced for the suppression of photosynthesis caused by salt stress.

In this study, nitrogen application could increase the content of osmoregulatory substances in rapeseed seedlings and improve osmotic adjustment. The Na^+^ content decreased and the K^+^ content increased with the increase of nitrogen application rate. This is consistent with the findings of Yang et al.^35^ It may be due to the fact that nitrogen application could reduce the uptake of Na^+^ by promoting the synthesis of plasma membrane-bound translocating proteins,^36^ resulting in a certain accumulation of K^+^. Ahanger et al.^17^ have shown that nitrogen application could increase the organic osmoregulatory substances (proline, sugars, and amino acids) in wheat seedlings, thereby increasing the tolerance to salt stress. However, in this study, the contents of SP and SS increased with the increase of nitrogen application rate, and the contents of SP and SS in the N_3_ treatments decreased compared with those in the N_2_ treatments. It may be due to the fact that nitrogen application could promote the synthesis of soluble protein and sugar.^37,38^ However, when nitrogen application is in excess, nitrogen assimilation is enhanced, and more carbon is used for the synthesis of nitrogen-containing compounds.^39^ Hao and Kang^40^ have also found that high application rate of nitrogen fertilizer could increase sucrose phosphate synthase and phosphoenolpyruvate (PEP) carboxylase, inhibit sugar synthesis, and reduce the contents of SP and SS. In this study, the content of Pro increased with the increase of nitrogen application rate, and the highest content of Pro was found in the N_3_ treatments. It may be due to that nitrogen application could increase the activity of P5C synthase during Pro synthesis and promote the synthesis of Pro.^41^

This study showed that nitrogen application could mitigate the toxic effects of excessive ROS production caused by salt stress by increasing the activity of key antioxidant enzymes and the content of non-enzymatic antioxidants. Nitrogen alleviates the oxidative stress caused by salt stress-induced excessive ROS by increasing the activity of key antioxidant enzymes and the content of non-enzymatic antioxidants. Our results showed that appropriate nitrogen application rate could enhance the activity of antioxidant enzymes by improving the expression of corresponding genes under salt stress.^42^ However, high nitrogen application rate (N_3_) decreased the activity. This may be due to that excessive nitrogen destroys the nutrient balance of rapeseed seedlings, resulting in the decrease of antioxidant enzyme activity.^43^ Besides, in this study, for non-enzymatic antioxidants, nitrogen application increased the accumulation of ASA to a certain extent under salt stress. This may be due to that nitrogen enhances the activities of enzymes (galactolactone dehydrogenase (GalLDH), monodehydroascorbate reductase (MDHAR), dehydroascorbate reductase (DHAR), and peroxidase (APX)) involved in the synthesis of ASA.^44^ At S_1_ and S_2_ levels, nitrogen application could regulate the activity of nitrate reductase and the content of nitric oxide to promote the synthesis of GSH.^45,46^ At S_3_ level, N_3_ treatment had the highest content of Car. This may be due to that nitrogen application increases the contents of rate limiting enzymes (PSY, PDS, and ZDS)^47,48^ in the process of Car synthesis, thus increasing the content of Car. Therefore, the application of moderate rate of N could effectively scavenge excess ROS through enhancing the activities of POD, SOD, and CAT in rapeseed seedlings at different levels of soil salinity. However, excessive nitrogen may lead to the increase in the content of non-enzymatic antioxidants (GSH and Car) to maintain the rate of ROS scavenging. Besides, in this study, Pro had a positive correlation with all enzymes (SOD, POD, and CAT) and non-enzymatic substances (ASA, GSH, and Car) of ROS scavenging system (*P* < .01). It may be due to that Pro could maintain osmotic balance, scavenge free radicals and singlet oxygen, stimulate antioxidant enzyme activity, and maintain the stability of proteins.^49^ SP had a positive correlation with antioxidant enzymes, GSH, and ASA (*P* < .01). It may be due to that SP has similar components to antioxidant enzymes and GSH, and ASA could promote the synthesis of SP.^50^ The negative correlation between K^+^ and antioxidant enzymes may be due to the fact that osmotic stress will occur when seedlings are subjected to oxidative stress, leading to the enhanced activity of antioxidant enzymes, aggravated osmotic stress, and decreased K^+^ content.^51^

Photosynthesis depends on a photosynthetic system composed of many complex proteins. Nitrogen, as a substrate for the synthesis of different proteins in the photosynthetic system, plays an important tole in photosynthesis.^52^ Therefore, the application of moderate rate of nitrogen in this study could decrease the negative effect of soil salinity on photosynthesis through increasing Chl content, P_n_, g_s_, and T_r_. However, photosynthesis was suppressed when nitrogen concentration was too high. It may be due to that nitrogen application could promote the synthesis of ribulose-1,5-bisphosphate(Rubisco) carboxylase and free amino acids, which reduces the nitrogen concentration in the components of electron transport chain and leads to the suppression of photosynthesis.^53^ Besides, it was found that the physiological indicators with a high correlation with photosynthesis, could further explain the indirect effect of nitrogen application on photosynthesis. Nitrogen is the basic element for synthesizing chlorophyll^54^ and plays a decisive role in plant photosynthesis^55^ In this study, the application of moderate rate of nitrogen could increase Chl content and promote photosynthesis. ROS scavenging and osmoregulatory system-related physiological indicators all could affect the photosynthesis in rapeseed seedlings, but the mechanisms differs. Application of moderate rate of nitrogen could increase the activities of antioxidant enzymes, which suppresses the chlorophyll breakdown caused by excessive ROS under salt stress.^56^ Nitrogen could promote the accumulation of ASA, a substance acting as a redox buffer in chloroplasts^57^ and playing a significant role in the protection of chloroplasts. Organic osmoregulatory substances SS and Pro could prevent the breakdown of chloroplast enzyme complex caused by excess Na^+^ in N_2_ treatment,^58^ increase the enzyme activity, K^+^ content, Rubisco activity,^59^ and PEP enzymes,^60^ and promote chlorophyll synthesis. Meanwhile, the application of moderate rate of nitrogen had a direct effect on P_n_, g_s_, and T_r_ by promoting the increase of SS and K^+^ content. It mainly due to that K^+^ could not only regulate stomatal opening and closing but also affect the activities of water channel proteins, thus affecting transpiration rate.^61^ SS is the initial product of dark reactions in photosynthesis. The increase in SS content could enhance respiration, stomatal opening, and transpiration rate.^39^ Therefore, the physiological responses in photosynthesis in rape seedlings under salt stress to nitrogen application is multifaceted, and the effects of different physiological indicators in the systems on photosynthesis are also crucial.

In summary, the application of moderate rate of nitrogen (N_2_) could enhance the tolerance of rapeseed seedlings to salt stress through synergistically regulating the osmoregulatory, ROS scavenging, and photosynthetic systems.

## 5. Conclusion

Nitrogen application could alleviate salt stress on the growth of rapeseed seedlings through increasing the content of organic osmoregulatory substances and K^+^. The application of moderate rate of nitrogen (0.30 g·kg^−1^) could obviously increase the antioxidant enzyme activity and the content of non-enzymatic substances to reduce the toxic effect of excessive ROS. Besides, nitrogen application could also increase the chlorophyll content to alleviate the damage of salt stress to photosynthesis. It should be noted that there are high correlations between Pro, SP and antioxidant enzymes, between antioxidant enzymes, ASA, pro and chlorophyll, between P_n_, g_s_, T_r_ and SS, K^+^, which jointly function in alleviating salt stress. This study not only improves the understanding of the mechanism of nitrogen application on alleviating the salt stress on rapeseed seedlings, but also provide a theoretical basis for the rational application of nitrogen in rapeseed cultivation in saline soils.

## Author statement

We have seen and approved the final version of the manuscript being submitted. We warrant that the article is our original work, has not received prior publication and is not under consideration for elsewhere.
